# In Contrast to Dietary Restriction, Application of Resveratrol in Mice Does not Alter Mouse Major Urinary Protein Expression

**DOI:** 10.3390/nu12030815

**Published:** 2020-03-19

**Authors:** Kathrin Pallauf, Ilka Günther, Dawn Chin, Gerald Rimbach

**Affiliations:** Institute of Human Nutrition and Food Science, University of Kiel, Hermann-Rodewald-Straße 6, 24118 Kiel, Germany

**Keywords:** glucocorticoid receptor, caloric restriction mimetic, longevity

## Abstract

Resveratrol (RSV) supplementation in mice has been discussed as partly mimicking the beneficial effects of dietary restriction (DR). However, data on putative benefits from resveratrol application in mice and other model organisms including humans is contradictory. Mouse major urinary proteins (MUPs) are a family of proteins that are expressed in rodent liver and secreted via urine. Impacting (mating) behavior and pheromone communication, they are severely down-regulated upon DR. We carried out two studies in C57BL/6Rj mice where RSV was either supplemented via diet or injected intraperitoneally for 8 weeks. Contrary to −40% DR, RSV did not decrease total MUP protein expression or *Mup* (amongst others *Mup3*, *Mup5*, *Mup6*, *Mup15*, and *Mup20*) mRNA levels in mouse liver when compared to ad-libitum (AL)-fed controls. Since inhibitory glucocorticoid response elements can be found in *Mup* promoters, we also measured glucocorticoid receptor (GR) levels in nuclear hepatic extracts. Consistent with differential MUP expression, we observed more nuclear GR in DR mice than in RSV-supplemented and AL control mice with no difference between RSV and AL. These findings point to the notion that, in mice, RSV does not mimic DR in terms of differential MUP expression.

## 1. Introduction

Mouse major urinary proteins (MUPs) are genes expressed in the liver (and to some extent in other tissues) of mice and excreted via urine [1]. As members of the lipocalin protein family, these gene products can function as carriers of pheromones, thereby stabilizing their olfactory potential [2,3]. Furthermore, MUPs can act as pheromones themselves [4] and regulate various behaviors such as aggression, ‘self-to-other’ signaling and mating [5]. Being androgen-induced, they are expressed at much higher levels in male mice [6] and positively affect fertility in female mice [7,8]. 

While MUPs are also found in some other mammals [9], humans only have a MUP pseudogene [4]. In mice, there are 21 MUPs and most of them are very similar to each other. It seems that mice express different but fixed subsets of MUPs depending on their strain [10] with the amount but probably not the ratio of excreted protein, depending on the circumstances [11]. 

Besides social regulation, restriction of dietary intake (DR) also affects MUP expression. In the liver of mice on DR, *Mup4*, *Mup5*, and *Mup1* transcription was decreased [12,13,14]. Additionally, *Mup1* was downregulated in adipose tissue [15] and the hypothalamus [16] of DR mice. Interestingly, this MUP family member was shown to regulate glucose metabolism, thereby increasing energy expenditure and glucose tolerance, and was downregulated in mice with genetically induced obesity such as db/db and ob/ob mice [17,18].

In laboratory rodents and other model organisms, DR has repeatedly prolonged lifespan [19]. In contrast, overfeeding shortens lifespan and leads to obesity in humans, promoting numerous life-shortening diseases [20,21]. In light of obesity-related pathologies increasing in prevalence [22] and the challenges related to restricting dietary intake [23,24], potential mimetics of dietary restriction (DRMs) that imitate the beneficial effects of DR without having to reduce energy consumption have been studied increasingly [25,26]. 

One of these putative DRMs is the secondary plant metabolite resveratrol (RSV). This 3,5,4′-trihydroxy-trans-stilbene is found in berries such as grapes (*vitis vinifera*) and other plants while its main dietary source is red wine [27]. Under certain circumstances, for example in mice on a high calorie diet, it prolonged lifespan [28]. Furthermore, RSV application may partially mimic the beneficial effects of dietary restriction in ageing mice [29]. Interestingly, RSV may also affect MUP expression, since gene transcription of *Mup1* and *Mup3* in RSV-supplemented mice was up-regulated [28].

Various pathways putatively regulating MUP expression have been described. Its upregulation can be promoted by application of testosterone with thyroxine or growth hormone [30], appears to be controlled epigenetically [31] and was shown to be increased by zinc fingers and homeoboxes2 (Zhx2) in mouse liver [32]. Additionally, under DR, binding of glucocorticoid receptor (GR) to GR elements in *Mup* promotors could contribute to decreased *Mup* transcription [13,33].

Since RSV is discussed as DRM and, in mice, MUPs are down-regulated upon DR (while RSV has been reported as up-regulating *Mups* [28]). We fed 4-month-old and injected 12-month-old C57BL/6Rj mice RSV for 8 weeks and compared their MUP expression to mice on an ad-libitum (AL) and DR diet. By doing so, we aimed at finding how MUP expression changed under RSV supplementation and whether is resembled changes induced by DR. Furthermore, we wanted to study DR- and RSV-induced changes in putative upstream regulators of MUP expression.

## 2. Materials and Methods 

### 2.1. Mice, Diet, and Intraperitoneal Injections

For the feeding trial, we purchased 12-week-old and, for the intraperitoneal (i.p.) trial, we purchased 11-month-old male C57BL/6Rj mice from Janvier Labs, Saint-Berthevin, France, housed them individually, and kept on a high fat, high sugar purified diet (18.7% protein, 21.1% fat, 13.4% starch, 32.9% sugar, 2.1 mg/kg cholesterol, Ssniff, Soest, Germany) from the moment they arrived at the animal facility as described previously [34,35]. After a 4 week adaptation period, the young and the old mice were divided into 3 groups each (*n* = 10 per group: non-supplemented *ad-libitum (*AL)*, −*40% DR, RSV-supplemented AL). DR was introduced gradually during 2 weeks to avoid digestive problems in the mice as described before [34,35]. When DR reached −40%, supplementation started and was carried out for 8 weeks. Mice of different ages were used since the i.p. trial was carried out prior to the feeding trial. In a first attempt to study RSV-induced changes in mice that may benefit ageing-related parameters, middle-aged mice and a dose that, considering allometric scaling [36], would be considered safe [37], were used. Since RSV application did not change mouse phenotype at a moderate dose of supplementation in 14 month old mice [35], we chose a dietary application at an approx. 3-fold higher dose in younger mice with the hope that younger mice may be more responsive towards RSV-application than older mice [34]. As reported before [34,35], we found RSV in the livers of the mice from the i.p. and the feeding trial but not in the corresponding AL control and DR mice when measuring RSV concentrations with UHPLC. For the injections, RSV (Carl Roth, Karlsruhe, Germany) was dissolved in PEG400 (Sigma-Aldrich Taufkirchen, Germany), at concentrations equimolar to 30 mg RSV/mL. This stock was diluted with saline (Braun, Melsungen, Germany) until reaching a PEG concentration of 20% v/v and injected at 24 mg RSV/kg bodyweight 3 times per week. The AL control and DR mice were injected with 20% PEG400 in saline [35]. 

For the dietary supplementation of RSV, one of the three feeding trial groups received the high-fat, high-sugar diet supplemented with RSV at 300 mg/kg diet for the first 4 weeks and then 400 mg/kg diet the following 4 weeks until sampling. By increasing the amount of RSV in the diet during the trial and while the animals were gaining weight, we had an average daily intake of 28 mg RSV/kg bodyweight throughout the trial [34]. Because of animal welfare laws, if individual DR mice lost too much weight, they were fed up to 70% of what the AL controls consumed [34,35].

Per experimental group, 8–10 mice were sacrificed at the end of the experiment. The experiment was approved according to German animal welfare laws (V 242-27717/2016 (46-4/16)). 

Further details on the experimental setup are shown in Table 1.

### 2.2. RNA Isolation and Quantitative Real-Time Polymerase Chain Reaction (qRT-PCR)

Total hepatic RNA was isolated using peqGOLD TriFastTM (Peqlab, Erlangen, Germany) following the manufacturer’s instructions. RNA concentrations and purity were determined with a Nanodrop 2000 (Thermo Fisher Scientific GmbH, Life Technologies, Darmstadt, Germany), adjusted to 100 ng/µL and stored at -80 °C. Gene expression levels were analyzed by one-step quantitative reverse transcriptase PCR using the SensiFAST™ SYBR No-ROX One-Step Kit (Bioline, Luckenwalde, Germany) with SybrGreen detection. PCR was performed in a Rotorgene 6000 cycler (Corbett Life Science, Sydney, Australia). Relative mRNA levels of target genes were normalized to the transcription of housekeeping gene *18S* and related to the mean of the AL control group set to be 1. Primer sequences are listed in Table 2.

### 2.3. Western Blotting

Cytosolic liver extracts were prepared by homogenizing fresh tissue in 10 mM HEPES (pH 7.9, 10 mM KCl, 1.5 mM MgCl2, 0.5 mM DTT, 0.1% Nonidet-P40, protease inhibitor cocktail and PhosSTOP^TM^, all Sigma-Aldrich) and leaving it on ice for 30 min with occasional vortexing before centrifuging at 4000 g and 4 °C for 5 min. With the supernatant containing the cytosolic proteins, the remaining pellet was resuspended in 40 mM HEPES (pH 7.9, 1 mM DTT, 400 mM KCl, 313 mM NaCl, 10% Glycerol, all Sigma-Aldrich), frozen at −80 °C and thawed on ice for extraction of the nuclear proteins. This suspension was incubated on ice for 30 min with occasional vortexing and centrifuged at 18000 g and 4 °C for 30 min in order to obtain the nuclear protein fraction in the supernatant. Protein concentrations were determined with the BCA assay (Thermo Fisher Scientific, Schwerte, Germany). The samples were mixed with loading buffer, denatured at 95 °C for 5 min, and separated in a SDS-PAGE on TGX Stain-Free Precast gradient gels in a vertical electrophoresis cell (all Biorad, Munich, Germany) using a buffer containing 0.025 M Tris base, 0.192 M glycine, and 0.1% SDS (all Sigma-Aldrich) and subsequently transferred onto a PVDF membrane. The membrane was blocked with 5% skim milk dissolved in tris-buffered saline (tris and sodium chloride from Roth) with 0.05% Tween-20 (VWR International, Darmstadt, Germany) and probed with a primary antibody overnight (cytosolic fraction: total MUP antibody sc-21856; nuclear fraction GR antibody, sc-393232, both Santa Cruz Biotechnology Inc., Heidelberg, Germany) followed by a secondary antibody (for MUP anti-goat sc-2354 fromSanta Cruz Biotechnology; for GR anti-mouse 1705047 from Biorad). The bands were visualized with ECL reagent (Thermo Fisher Scientific) in a ChemiDoc XRS system (BioRad). 

Densitometries were carried out using Image Lab 5.0 (BioRad) and normalizing to total protein.

### 2.4. Statistics

Statistical analyses were performed using the software R version 3.4.3. [38]. Data evaluation started with the definition of an appropriate model. For the PCRs, this was a linear mixed model with PCR run as a random effect. For Western blot densitometries, we included the blot as random effect, unless it was negligible for explaining the data [39,40]. Based on a graphical residual analysis, the data were assumed to be approximately normally distributed. When appearing as normally distributed, an analysis of variances (ANOVA), followed by multiple contrast tests (Tukey) [41] were conducted.

## 3. Results

### 3.1. Food Intake and BW Do Not Differ Between Non-supplemented and RSV-Supplemented Mice

As reported before [34,35], RSV-supplemented mice did not significantly differ in final bodyweight (BW) from non-supplemented AL control mice (CON). For the i.p. mice, BWs were (+/− standard deviation(SD)) CON 40.8 +/− 6.1 g versus RSV 41.2 +/− 4.1 g and for the mice from the feeding study CON 37.4 +/− 3.9 g versus RSV 35.2 +/− 2.5 g. The DR mice had much lower BWs, 25.7 +/− 1.5 g, and 21.1 +/− 1.3 g in the i.p. and feeding study, respectively. Feed intake for the mice consuming RSV or CON diets did not differ between the groups and was 3.0 g/ day for the i.p. mice and 2.7 g/day for the mice supplemented via diet.

### 3.2. Compared to a Considerable MUP Protein Level Decrease in CR Mice, MUP Levels between RSV Mice and the AL Control Appear Similar

**Figure 1:**
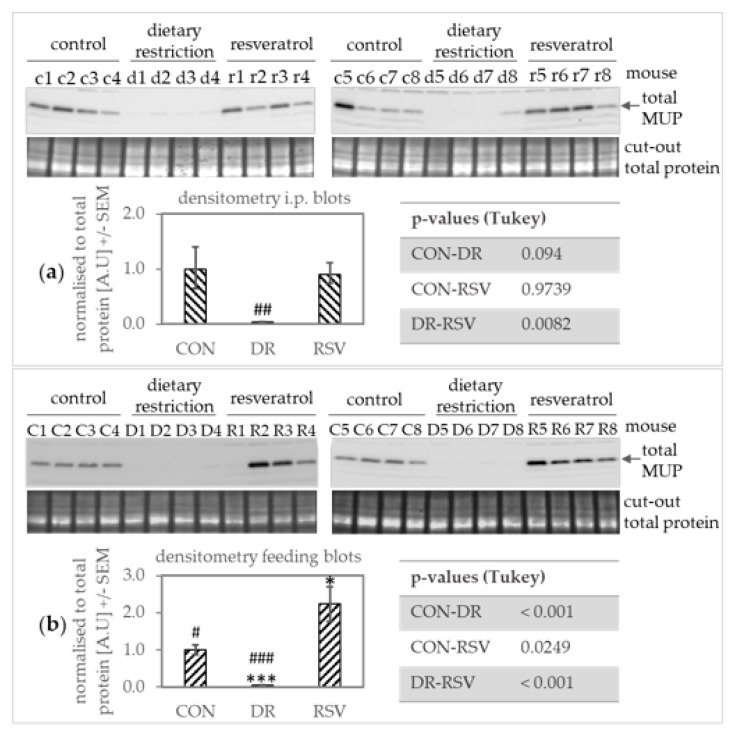
Western blots showing mouse urinary protein (MUP) levels in livers of mice supplemented with resveratrol (RSV) compared to ad-libitum control mice (CON) and mice under dietary restriction (DR). (**a**) RSV was injected intraperitoneally, (**b**) RSV was supplemented via diet. MUP levels fall drastically upon dietary restriction but not so by RSV application. Liver lysates from 12 different mice per blot were run, with two blots per experiment this is *n* = 8 mice per group. Mice from the i.p trial were named with a lower key letter (c—AL control; d—dietary restriction, r—resveratrol), animals from the feeding trial were named with the corresponding upper key letter (C—AL control, D—dietary restriction, R—resveratrol). The number refers to the number given the animal in the trial. Data is presented as mean +/− SEM. * *p* < 0.05, *** *p* < 0.001 compared to CON; # *p* < 0.05, ## *p* < 0.01, ### *p* < 0.001 compared to RSV (Tukey).

While there appears to be a dramatic reduction in MUP expression in DR mice, injecting RSV did not change MUP protein levels significantly. In mice fed RSV, there may be a slight increase in MUP protein. However, in DR mice, one hardly sees a MUP band in the Western blot (Figure 1). In CON and RSV mice, MUP levels vary within the groups. Furthermore, due to high similarity between MUPs 1-21 [9], the antibody can only recognize total MUP protein.

### 3.3. Mup mRNA Levels Seem Unaffected by RSV Supplementation but are Decreased by DR

To further study possible differences in MUP expression caused by RSV and DR, we measured gene transcription for various *Mups*. Once again, the high similarity between the Mup family members made it impossible to distinguish between all of them. However, we were able to design specific primers for *Mup3*, *Mup5*, *Mup6*, *Mup15*, and *Mup20*. Because of its possible implication in glucose metabolism regulation, we were also interested in Mup1 expression. Unfortunately, we could not design a primer that would solely amplify the three *Mup1* transcripts (Table 1). We normalized mRNA levels to ribosomal *18S* levels, since other housekeeping genes typically used in mouse experiments (i.e., beta-2 microglobulin NCBI-ID 12010, hydroxymethylbilane synthase NCBI-ID 15288 [42]) appeared to be regulated by DR. 

**Figure 2:**
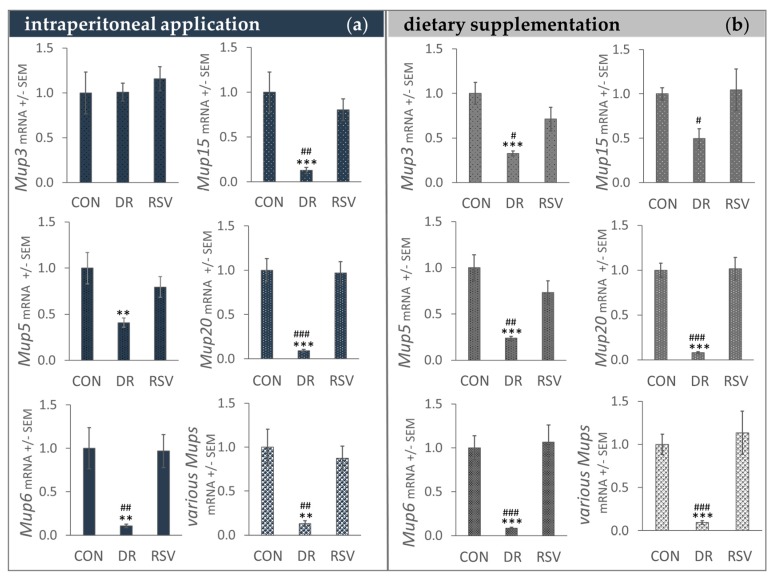
Real-time RT PCR results for gene coding for mouse urinary protein (*Mup*) mRNA levels in livers of mice injected intraperitoneally (**a**) or fed (**b**) with resveratrol (RSV) compared to ad-libitum control mice (CON) and mice under dietary restriction (DR). mRNA levels were normalized to *18S* ribosomal RNA and related to CON which was set to be 1. Data is shown as means +/− SEM. ** p < 0.01, *** *p* < 0.001 compared to CON; # *p* < 0.05, ## *p* < 0.01, ### *p* < 0.001 compared to RSV (Tukey).

Feeding RSV at a daily dose of approx. 28 mg / kg bodyweight or injecting RSV at 24 mg/ kg bodyweight 3 times per week equaling approx. 10 mg/day per kg bodyweight for 8 weeks did not affect Mup transcription (Figure 2). 

However, in the feeding trial, DR reduced *Mup3, Mup5, Mup6, Mup2*0, and possibly *Mup1* transcription dramatically compared to AL control mice. *Mup15* transcription may also be reduced, the p-value for comparing DR with CON mice showing a trend (0.0722). Interestingly, in DR mice from the i.p. trial, *Mup3* was not downregulated while the other *Mups* measured in those mice showed significantly lower mRNA levels compared to CON and RSV mice (Figure 2).

### 3.4. Translocation of the Glucocorticoid Receptor into the Nucleus May be Increased Under DR but not RSV Supplementation

Since ZHX2, IGF1, and GR may regulate MUP expression [13,30,32], we measured Zhx2 mRNA and protein levels, *Igf1* mRNA levels and GR translocation into the nucleus in the liver of our mice. 

*Zhx2* mRNA did not differ significantly between DR and other mice but showed a slight increase when comparing RSV-fed (not injected) with the corresponding CON mice (*p* = 0.0256, Supplementary Figure S1). *Igf1* mRNA appeared to increase upon DR (p-values for differences in *Igf1* mRNA levels for i.p. trial: C-DR = 0.0004, DR-RSV = 0.0010, RSV-C 0.8436; feeding trial: C-DR = 0.0709, DR-RSV = 0.0011, RSV-C = 0.2357; Supplementary Figure S1).

Higher GR protein levels in mice on DR can be seen and are confirmed by densitometry in Western blots from the nuclear fractions. However, it needs to be pointed out that separating the cytosolic fraction from the nuclear fraction by using different buffers yields less pure fraction than more sophisticated methods. Yet, since all samples were treated the same, the Western blot analysis of this fraction may add some information on nuclear protein levels.

## 4. Discussion

In accordance with previous findings [12,13,14,43], in our male C57BL/6 on a high fat diet, Mup mRNA and protein expression were decreased (except for *Mup3* transcription in the i.p. trial, Figure 1; Figure 2). Down-regulation of Mup under DR has been reported for different levels of restricting dietary intake (−10%, −20%, −30%, and −40% [44]) as well as different diets such as a standard diet with 10% of energy from fat [44] and a high fat diet with 42% of energy from fat [43]. As mentioned in the introduction, DR has repeatedly prolonged lifespan in model organisms [45] and RSV also promoted longevity in some experimental settings [46]. Furthermore, RSV somewhat mimicked transcriptional changes observed under DR in mice [47,48]. However, while RSV-supplemented mice on a high fat diet lived longer [28], mice on a standard diet did not [48] and RSV supplementation trials render inconclusive data [49,50]. Since Mups appear consistently down-regulated upon DR, studying their expression in RSV-supplemented mice could somewhat help elucidating to which extent RSV may mimic DR. In contrast to DR, RSV did not decrease Mup expression (Figure 1; Figure 2). To the best of our knowledge, Mup down-regulation under RSV has not been observed. Interestingly, in the male C57BL/6 mice on a high fat diet that lived longer upon RSV-supplementation, liver whole-genome-microarrays showed transcriptional *Mup1* and *Mup3* up-regulation [28]. *Mup* up-regulation could explain the small increase in MUP protein levels we observed in our male C57BL/6 on a high fat diet Figure 1b. Yet, we could not find a specific primer for *Mup1*, and thus putative *Mup1* upregulation could have been masked by the transcription of other *Mups* also measured. Furthermore, our i.p. mice had received RSV at a lower dose (approx. 10 mg versus 22 mg/ day per kg bodyweight) and all mice for a shorter time (8 weeks versus 6 months) than the mice in Baur et al. [28]. 

Besides dose and application route, mice from the i.p. and feeding trial differed in age since the i.p. trial mice were older than the feeding trial mice (approx. 1 year versus 18 weeks at the beginning of the intervention). While the results for Mup expression were similar in both studies, we found a difference between trials when measuring *Mup3* mRNA levels in DR compared to CON mice. Here, only the younger mice from the feeding trial responded to DR feeding. In the older mice, *Mup3* transcription seemed unchanged (Figure 2). Possibly, younger mice respond better to changes in their dietary regimen. It is interesting to note that high dose RSV application had a small effect on body composition and insulin sensitivity in the younger mice while we did not see such an effect in the older mice using a lower dose [34,35].

Remarkably, transcription of *Zhx2,* which, in a publication by Jiang [32], positively regulated transcription of various *Mups* including *Mup20*, was slightly increased in RSV-fed mice (*p*-value for CON-RSV 0.0256, Supplementary Figure S1). However, changes in *Zhx2* did not coincide with changes in *Mup* mRNA levels. In DR mice from the feeding trial, lower than CON *Mup* mRNA levels were accompanied with unchanged (if not increased *p* = 0.2218) *Zhx* levels and *Mup20* levels found in livers of CON and RSV mice showed no difference (*p* = 0.99) (Figure 2). 

Based on the finding that MUP expression is favored by GH [30], we hypothesized that downstream *Igf1* transcription may reflect the changes observed for *Mup* RNA levels. Surprisingly, *Igf1* mRNA levels were increased in our DR mice. It has been reported that neither dietary restriction nor age had an effect on *Igf1* mRNA levels in the C3B10RF1 mice from Spindler et al. [51], however, IGF1 protein was reduced in C57BL/6 mice on DR [44]. Of interest, Spindler’s mice [51] and Mitchell’s mice [44] were on diets with approximately 3%–5% fat while our mice were on a diet with 21.1% fat. Thus, the diets might have contributed to different IGF1 levels. Additionally, as well as its upstream regulators, *Igf1* mRNA and protein levels are subjected to circadian rhythm [52]. DR feeding disturbs this circadian rhythm [53] and we only have mRNA levels from one timepoint. Therefore, it is possible that *Igf1* mRNA levels in our DR mice are increased because of the timepoint used and not because mice on DR generally express more Igf1 than their AL controls. 

**Figure 3:**
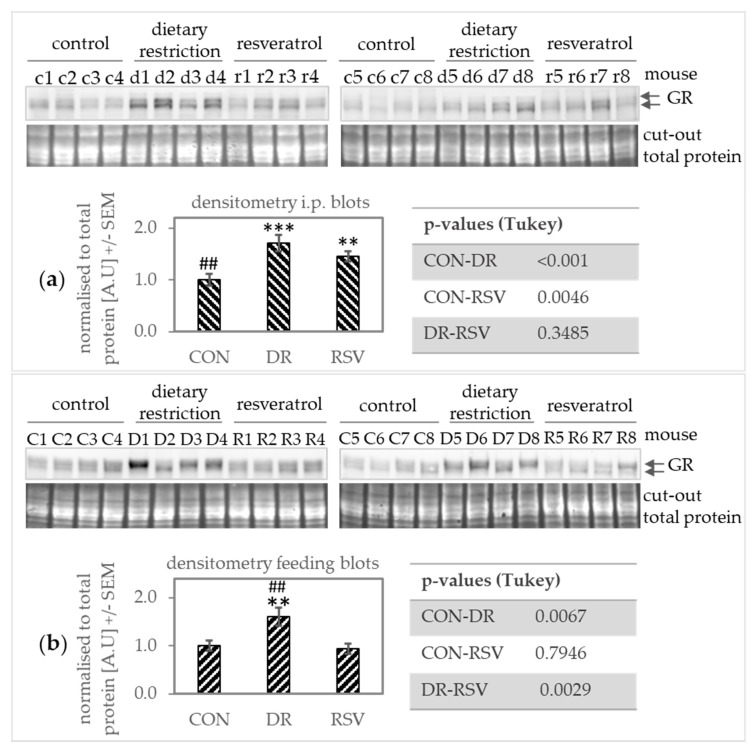
Western blots showing glucocorticoid receptor (GR) levels (double band) in the nuclear fraction of hepatic protein samples from mice supplemented with resveratrol (RSV) compared to ad-libitum control mice (CON) and mice under dietary restriction (CR). (**a**) RSV was injected intraperitoneally, (**b**) RSV was supplemented via diet. GR levels in the nucleus appear to rise upon dietary restriction. Mice from the i.p trial were named with a lower key letter (c—AL control; d—dietary restriction, r—resveratrol), animals from the feeding trial were named with the corresponding upper key letter (C—AL control, D—dietary restriction, R—resveratrol). The number refers to the number given the animal in the trial. Data is presented as mean +/− SEM. ** *p* < 0.01 *** *p* < 0.001 compared to CON; ## *p* < 0.01 compared to RSV (Tukey).

In contrast, we found that GR translocation to the nucleus (Figure 3) coincided better with the pattern of Mup expression than *Zhx2* or *Igf1* measurements. Giller et al. [13] found inhibitory response elements for the GR in the promotor of *Mup5*. Therefore, diminished Mup levels in DR mice could have been caused, in part, by GR activation. On the one hand, GR activation under DR is consistent with DR being a stressful challenge for the mice and glucocorticoids acting anti-reproductively [54]. On the other hand, the artificial ligand of GR dexamethasone inhibited decrease of *Mup* RNA levels in an ex vivo approach using hepatocytes from BALB/c mice [55]. To further characterize the importance of GR signaling for Mup expression, gene silencing experiments should be carried out. Unfortunately, such studies were beyond the scope of our study.

While RSV has been referred to as putative DRM, its impact on Mup regulation shows that RSV, at least under certain circumstances, does not mimic DR. In humans, no Mup is expressed since humans only have a pseudogene [4]. However, upstream signaling pathways and hormones such as testosterone, thyroxine, or growth hormone that were described as controlling Mup expression [30] also exist in humans. Yet, it has been hypothesized that animals such as mice, which live in environments with fluctuating food supply and adjust their fertility accordingly, may benefit to a stronger extent from DR than organisms that have a more regular food supply such as humans [56]. While mice surely have their limitations for studying human longevity, review of lifespan interventions in mice renders heterogeneous data pointing to the notion that RSV may not be a DRM, which reflects outcomes in human trials [57].

## 5. Conclusions

While restricting dietary intake in C57BL/6 mice on a high calorie diet drastically reduces Mup gene and protein expression, dietary or intraperitoneal supplementation of RSV does not affect Mup levels when compared to non-supplemented AL controls. Thus, in this experimental setup and in terms of differential MUP expression, RSV does not act as a DRM.

## Figures and Tables

**Table 1:** Experimental design used showing how the three groups per trial (ad libitum (AL) control without resveratrol (RSV) application, RSV application group and −40% DR group) were treated and what / how many mice were used. Dietary restriction (DR) was introduced gradually and mice were subjected to a six-week adaptation period on the high fat, high sugar diet before beginning the trial [34,35].

Male C57BL/6Rj	Group *n* = 8–10	Diet High Fat, High Sugar	Injections (PEG/Saline)	RSV Dose and Duration of Application	Age (Start of RSV Supplementation)
**intra-peritoneal (i.p) trial**	AL control	non supplemented AL	3*/week	none	≈1 year
	RSV application	non supplemented AL	3*/week containing RSV	≈10 mg/kg bodyweight/ day for 8 weeks	
	DR	non supplemented −40% DR	3*/week	none	
**feeding trial**	AL control	non supplemented AL	none	none	≈18 weeks
	RSV application	RSV supplemented AL	none	≈28 mg/kg bodyweight/ day for 8 weeks	
	DR	non supplemented −40% DR	none	none	

**Table 2:** Primers used for qRT-PCR.

Gene Name	NCBI Gene ID	forward Sequence	reverse Sequence	Other Targets/Remarks
*Mup1 (various Mups)*	17840	GAAGCTAGTTCTACGGGAAGGA	AGGCCAGGATAATAGTATGCCA	*Mup 2,7,8,9,10,* *11,12,13,14,17,19*
*Mup3*	17842	TTGGTTTTCTATTGCTGAAGCCT	CCAATCGCAGTCATTTCGGTG	
*Mup5*	17844	ATGGAGCTCTTTGGTCGA	TGTATGGAAGGGAAGGGATG	
*Mup6*	620807	TTCCAGCTGATGTCGCTCTA	GCGATTGGTTTTGGTGAAGT	
*Mup15*	100039150	GTGGAGTGTAGCCACGATCA	CAGCAGCAACAGCATCTTCA	
*Mup20*	381530	ATGAAGCTGCTGGTGCTG	TTGTCAGTGGCCAGCATAATAG	
*18S*	19791	GGTAACCCGTTGAACCCCAT	CAACGCAAGCTTATGACCCG	
*Zhx2*	387609	GAGCCAGCAGAGTTCCATTT	GCAATCTCTGAGCGAACCAG	in supplements
*Igf1*	16000	TGGATGCTCTTCAGTTCGTG	GCAACACTCATCCACAATGC	in supplements

## Supplementary Materials

The following are available online at https://www.mdpi.com/2072-6643/12/3/815/s1. Figure S1: real-time RT PCR results for *Igf1 and Zhx2* mRNA levels in livers of mice fed (a) or injected intraperitoneally (b) with resveratrol (RSV) compared to *ad-libitum* control mice (CON) and mice under dietary restriction (DR).
