# Supplementary figures and images for: In Contrast to Dietary Restriction, Application of Resveratrol in Mice Does not Alter Mouse Major Urinary Protein Expression
Source: Nutrients. 2020 Mar 19;12(3):815. doi: 10.3390/nu12030815 (PMC7146287; doi:10.3390/nu12030815)

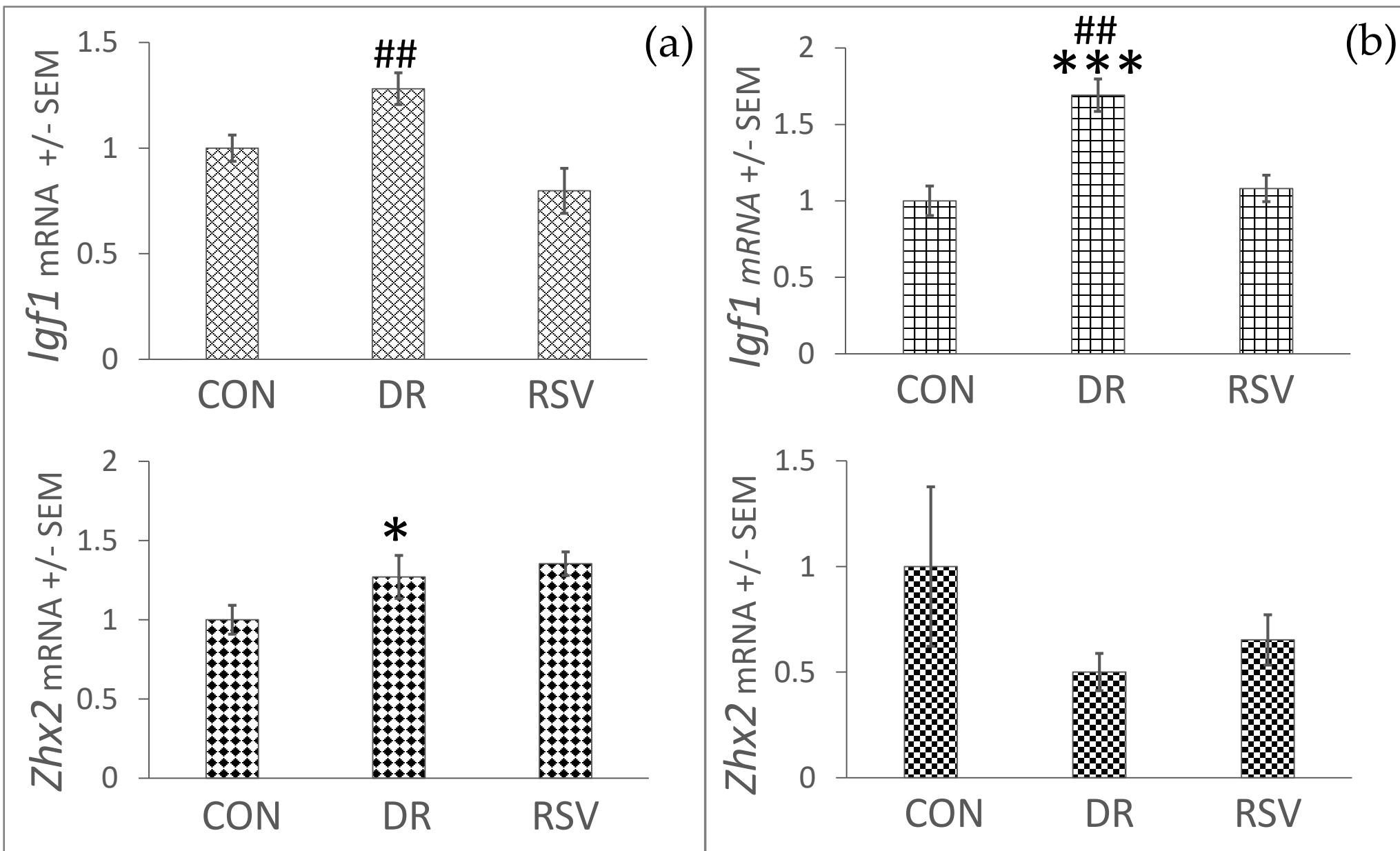

feeding

ip

Supplement: Supplementary file 1 [file nutrients-12-00815-s001.pdf]
